# The Instant Effects of Continuous Transcutaneous Auricular Vagus Nerve Stimulation at Acupoints on the Functional Connectivity of Amygdala in Migraine without Aura: A Preliminary Study

**DOI:** 10.1155/2020/8870589

**Published:** 2020-12-10

**Authors:** Wenting Luo, Yue Zhang, Zhaoxian Yan, Xian Liu, Xiaoyan Hou, Weicui Chen, Yongsong Ye, Hui Li, Bo Liu

**Affiliations:** ^1^Department of Radiology, The Second Affiliated Hospital of Guangzhou University of Chinese Medicine, Guangzhou 510120, China; ^2^The Second Clinical College, Guangzhou University of Chinese Medicine, Guangzhou 510405, China; ^3^Department of Neurology, The Second Affiliated Hospital of Guangzhou University of Chinese Medicine, Guangzhou 510120, China

## Abstract

**Background:**

A growing body of evidence suggests that both auricular acupuncture and transcutaneous auricular vagus nerve stimulation (taVNS) can induce antinociception and relieve symptoms of migraine. However, their instant effects and central treatment mechanism remain unclear. Many studies proved that the amygdalae play a vital role not only in emotion modulation but also in pain processing. In this study, we investigated the modulation effects of continuous taVNS at acupoints on the FC of the bilateral amygdalae in MwoA.

**Methods:**

Thirty episodic migraineurs were recruited for the single-blind, crossover functional magnetic resonance imaging (fMRI) study. Each participant attended two kinds of eight-minute stimulations, taVNS and sham-taVNS (staVNS), separated by seven days in random order. Finally, 27 of them were included in the analysis of seed-to-voxel FC with the left/right amygdala as seeds.

**Results:**

Compared with staVNS, the FC decreased during taVNS between the left amygdala and left middle frontal gyrus (MFG), left dorsolateral superior frontal gyrus, right supplementary motor area (SMA), bilateral paracentral lobules, bilateral postcingulum gyrus, and right frontal superior medial gyrus, so did the FC of the right amygdala and left MFG. A significant positive correlation was observed between the FC of the left amygdala and right SMA and the frequency/total time of migraine attacks during the preceding four weeks.

**Conclusion:**

Continuous taVNS at acupoints can modulate the FC between the bilateral amygdalae and pain-related brain regions in MwoA, involving the limbic system, default mode network, and pain matrix, with obvious differences between the left amygdala and the right amygdala. The taVNS may produce treatment effects by modulating the abnormal FC of the amygdala and pain networks, possibly having the same central mechanism as auricular acupuncture.

## 1. Introduction

Migraine without aura (MwoA), the most common type of migraine, is the second-largest neurological disorder affecting the global disability-adjusted lifespan year, causing a severe burden on the health system and budgets [1]. However, its conventional medical treatments for migraines are far from satisfactory [2]. It is essential to find more effective and safe treatments.

A recent study [3] suggested that the occurrence of migraine was related to the imbalance of the autonomic nervous system, and increasing parasympathetic (mainly including vagal) nerve activity could alleviate the symptoms of migraine [4]. However, there are only a few indirect ways to regulate the vagal nerve tension, such as breathing regulation and yoga [5]. It is worth noting that noninvasive vagus nerve stimulation (nVNS) can directly regulate vagal nerve activity to treat migraines effectively [6, 7]. It has been approved for treating episodic migraines by the Food and Drug Administration (FDA) of the USA in 2017, reducing the severity and frequency of headache [8, 9], improving the quality of life and reducing the cost of treatment [10], and being well-tolerated [11]. Notably, transcutaneous auricular vagus nerve stimulation (taVNS), a kind of simpler and effective nVNS [12], may have the same treatment effect and mechanism as auricular acupuncture [13]. A growing body of evidence suggests that both auricular acupuncture and taVNS can induce antinociception and relieve symptoms of migraine. However, the instant effects and central mechanism of electrical stimulation at auricular points innervated by the vagal nerve in MwoA are still unclear.

Previous neuroimaging evidences [14–17] suggested that migraine was associated with abnormal structure and function of brain regions involved in pain and emotional processing, such as the prefrontal cortex (PFC), cingulate gyrus, supplementary motor area (SMA), amygdala, hippocampus, insula, precuneus, periaqueductal gray matter (PAG), thalamus, cerebellum, and et al. Among them, the vital functions of the amygdalae in migraine have already attracted many researchers' attentions [18, 19]. Recent studies [20–22] demonstrated that the amygdalae were also involved in pain processing and modulation. A neuroimaging study found that the amygdala was activated during the headache, and its decreased volume was related to the frequency of headache attacks [23]. The FC of the left amygdala and left middle cingulate gyrus increased in episodic migraine compared with healthy controls, and there were obvious differences between the left amygdala and the right amygdala [24]. These results suggested that the dysfunction of the amygdala might be a potential mechanism of MwoA. However, little has known about the FC of the bilateral amygdalae and other regions in MwoA patients before and during taVNS so far.

Based on the above argument, we hypothesized that continuous taVNS at auricular points could modulate the abnormal FC of the left/right amygdala in MwoA patients. Then, the voxel-wise FC was analyzed during continuous stimulation, using the left/right amygdala as seeds, respectively.

## 2. Materials and Methods

### 2.1. Participants

Thirty episodic migraineurs without aura were recruited for the single-blind, crossover functional magnetic resonance imaging (fMRI) study from the neurology clinic outpatient of the Second Affiliated Hospital of Guangzhou University of Chinese Medicine from January to December in 2018. Each participant attended two fMRI scan sessions during the eight-minute continuous stimulation at auricular points separated by seven days, one for taVNS and another for sham-taVNS in random order. Informed consent was obtained from all participants. This study protocol was approved by the Institutional Review Board of the Second Affiliated Hospital of Guangzhou University of Chinese Medicine.

Similar to our previous study [25], the diagnosis of migraine was based on the International Classification of Headache Disorders 2^nd^ Edition (ICHD-II) [21, 26], as diagnosed by a specialist working at the neurology outpatient service.

The inclusion criteria were as follows: (1) aged 18-45 years old, (2) right-handed, (3) at least six months of migraine duration, (4) having at least one attack per month, (5) having no prophylactic headache medicine during the past one month, and (6) having no psychoactive or vasoactive drugs during the past three months.

The participants were excluded if any of the following criteria were met: (1) was caused by other diseases or special types of migraine, (2) attacked within 48 hours before and during the scan, (3) had pregnancy or lactation, (4) had severe head deformity or intracranial lesions, (5) had other chronic pain diseases, and (6) had the standard score of self-rating anxiety or depression scale greater than 50.

### 2.2. Intervention Program

#### 2.2.1. Stimulator

Huatuo auricular vagus nerve stimulators were from SDZ-II, Suzhou Medical Appliance Factory, China.

#### 2.2.2. Stimulation Parameters

**Figure 1:**
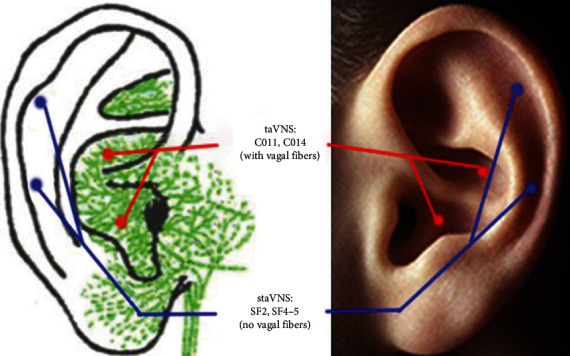
The innervations of the auricular branch of the vagal nerve and the points stimulated at the ear. The vagal nerve branch is marked by green color, the taVNS points by red, and the staVNS points by blue.

The stimulation parameters are as follows: constant voltage, continuous wave, 1 Hz, 0.2 ms, and the current intensity below the pain threshold, lasting 8 minutes. taVNS points: CO11 and CO14, left cymba concha (abundant in vagal afferent fibers). StaVNS points: SF2 and SF4-5, left scapha (no vagal afferent fibers) (Figure 1) [25, 27].

### 2.3. Clinical Assessments

The demographic information and clinical scale data of all patients were collected, including Migraine Specific Quality-of-Life Questionnaire (MSQ), Self-rating Depression Scale (SDS), Self-rating Anxiety Scale (SAS), Visual Analog Scale (VAS), the frequency, and total duration time of migraine attacks during the past four weeks preceding the fMRI scans.

### 2.4. MRI Data Acquisition

All MRI scans were conducted on a 3.0T Siemens MRI scanner (Siemens MAGNETOM Verio 3.0T, Erlangen, Germany) with a 24-channel phased-array head coil. All subjects were told to stay awake and remained motionless during the scan, keeping their eyes closed. Each scan session lasted approximately 20 minutes. The orders of MRI scans were as follows: a high-resolution anatomical image (MPRAGE), an eight-minute resting-state functional MRI, and an eight-minute continuous real or sham-taVNS (fMRI was applied during this continuous stimulation period).

T1-weighted MPRAGE was applied with the following parameters: TR = 1900 ms, TE = 2.27 ms, FOV = 256 mm × 256 mm, flip angle = 9°, matrix = 256 × 256, thickness = 1.0 mm, and 176 slices. The eight-minute resting state and eight-minute continuous real or sham-taVNS fMRI scan were acquired with the following parameters: TR = 2000 ms, TE = 30 ms, FOV = 224 mm × 224 mm, flip angle = 90°, matrix = 64 × 64, thickness = 3.5 mm, 31 slices, and 240 time points.

### 2.5. fMRI Data Analysis

#### 2.5.1. fMRI Data Preprocessing

The fMRI data preprocessing was performed using DPABI 3.0 and SPM 12.0 based on MATLAB (The MathWorks, Natick, MA, USA). After removing the first ten volumes of each participant, the functional images were corrected for the intravolume acquisition time delay using slice timing and realignment. One of the participants was excluded based on the criteria of displacement > 2.5 mm or angular rotation > 2.5° in any direction. Then, all corrected functional data were normalized to the Montreal Neurological Institute (MNI) space and resampled to a 3 mm isotropic resolution. The resulting images were further temporally band-pass filtered (0.01–0.08 Hz) to remove the effects of low-frequency drift and high-frequency physiological noise. Finally, 24 head-motion parameters, white matter signals, and cerebrospinal fluid signals were regressed using a general linear model, and linear trends were removed from the fMRI data. Spatial smoothing was also performed before the functional connection analysis using a Gaussian filter (6 mm full-width half maximum; full width at half maximum [FWHM]).

#### 2.5.2. Seed-to-Voxel FC Analysis

**Figure 2:**
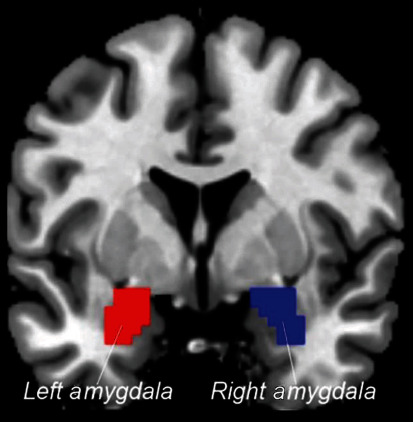
The left and right amygdalae were, respectively, generated based on the AAL template as seeds. Red: the left amygdala; blue: the right amygdala.

The seeds of the left amygdala and right amygdala were defined separately, using the AAL template (Figure 2) (same to the previous literature [24]). Then, the amygdalae were resliced with the brain mask template with 63 × 71 × 63 size. The FC calculation was performed in DPABI (V3.0). The averaged time courses of the left/right amygdala were extracted, respectively. Next, Pearson correlation was used to calculate the FC between the extracted time courses and the time courses of the whole brain in a voxel-wise manner, respectively. The correlation coefficient map was then converted into a Fisher-Z map by Fisher's *r*-to-*z* transformation to improve normality.

### 2.6. Statistical Analysis

The intergroup analysis (taVNS vs. staVNS) of the FC was performed using a paired *t*-test, with the mean head-motion value (mean FD Jenkinson) as covariate variables. A threshold of voxel-wise *p* uncorrected and cluster-level *p* corrected by familywise error corrected (FWE) were applied for multiple comparison correction. If voxel-wise *p* < 0.001 and cluster-level *p* < 0.05, the difference was statistically significant.

Besides, in the baseline resting state and continuous stimulation state, we, respectively, extracted the average *z* values of significantly altered clusters of the left/right amygdala (taVNS vs. staVNS). Then, the differences of the *z*FC values were compared using a paired *t*-test between taVNS and staVNS in the two states, and *p* < 0.05 was considered to be statistically significant [28]. We also explored the association between the initial clinical assessments and the altered *z*FC values (taVNS minus staVNS) in continuous stimulation state across all subjects after Bonferroni correction.

## 3. Results

### 3.1. Clinical Results

Twenty-seven patients completed the study and were included in the data analysis, because the two participants did not finish the fMRI scan, and one was excluded for displacement > 2.5 mm or angular rotation > 2.5° in any direction. The demographics are shown in Table 1.

### 3.2. fMRI Results

#### 3.2.1. Left Amygdala as Seed

**Figure 3:**
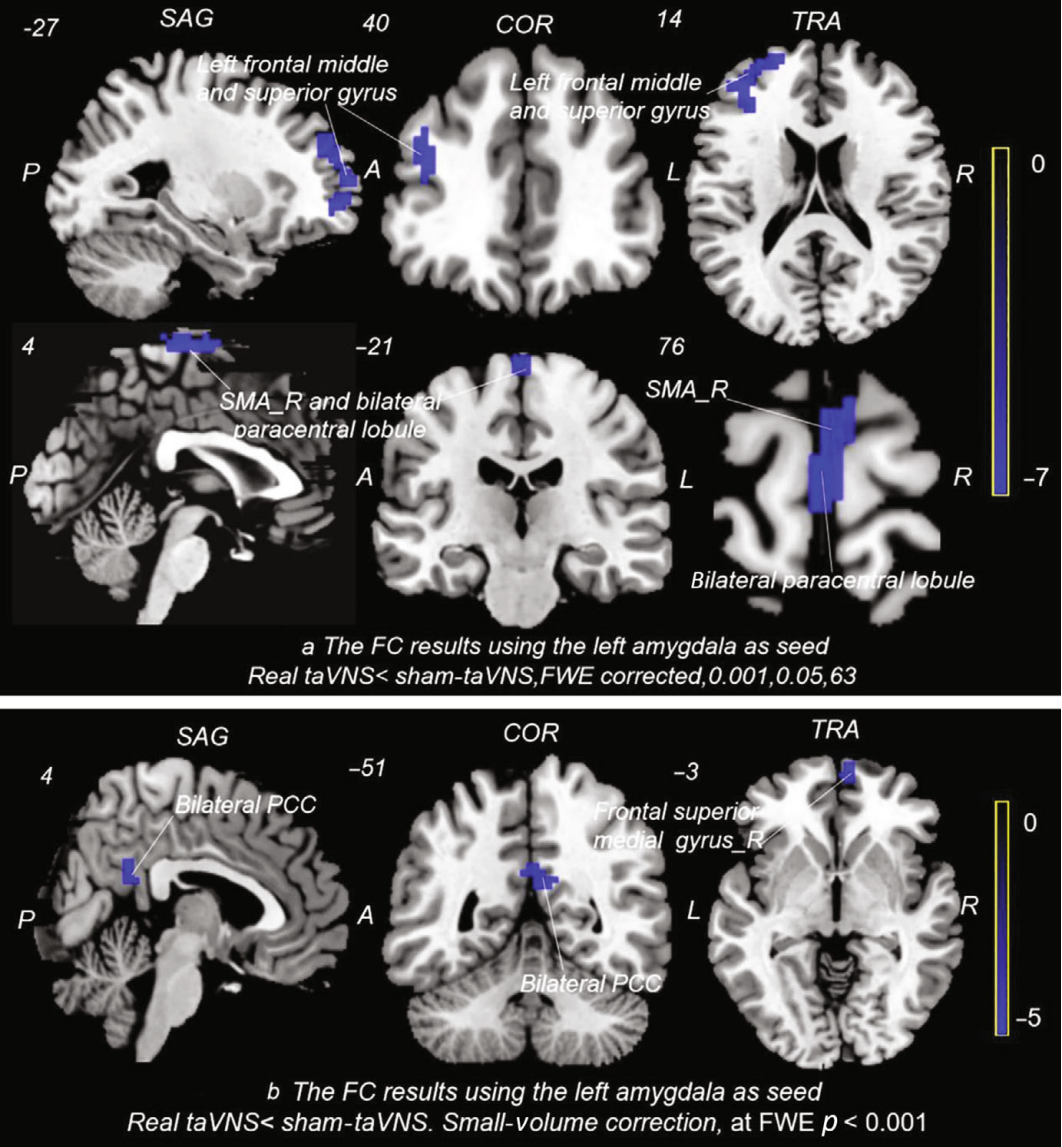
The functional connectivity (FC) of the left amygdala in MwoA during continuous stimulation (taVNS vs. staVNS). L: left; R: right; P: posterior; A: anterior; SAG: sagittal; COR: coronal; TRA: transverse; PCC: postcingulum gyrus; SMA: supplementary motor area; FWE: familywise error correction. Compared with sham-taVNS, the FC significantly decreased between the left amygdala and left frontal middle gyrus, left frontal superior gyrus, right SMA, bilateral paracentral lobule ((a) FWE multiple comparison correction, voxel-level *p* < 0.001 and cluster-level *p* < 0.05), bilateral PCC, and right frontal superior medial gyrus ((b) small-volume correction, FWE *p* < 0.001) in taVNS.

The brain regions with decreased FC of the left amygdala mainly located in the left middle frontal gyrus (MFG), left dorsolateral superior frontal gyrus (SFG), right supplementary motor area (SMA), and bilateral paracentral lobule during continuous stimulation of taVNS compared with sham-taVNS (Table 2 and Figure 3(a)). No significant increased FC was observed.

Since the postcingulum cortex (PCC) and frontal medial gyrus are important nodes in the default mode network (DMN) [29, 30] and endogenous pain-inhibitory circuits [31, 32]. They play a very important role in the migraine pathogenesis [29–32]. Similar to the previous study [33], the small volume correction with a threshold of voxel-wise *p* < 0.001 and cluster-level *p* < 0.05 was used in ROI analysis. The direct intergroup comparison revealed more decreased FC in taVNS compared with the sham, in the bilateral PCC and the right frontal superior medial gyrus (small-volume corrected at *p*_FWE_ < 0.001) (Table 2 and Figure 3(b)).

**Figure 4:**
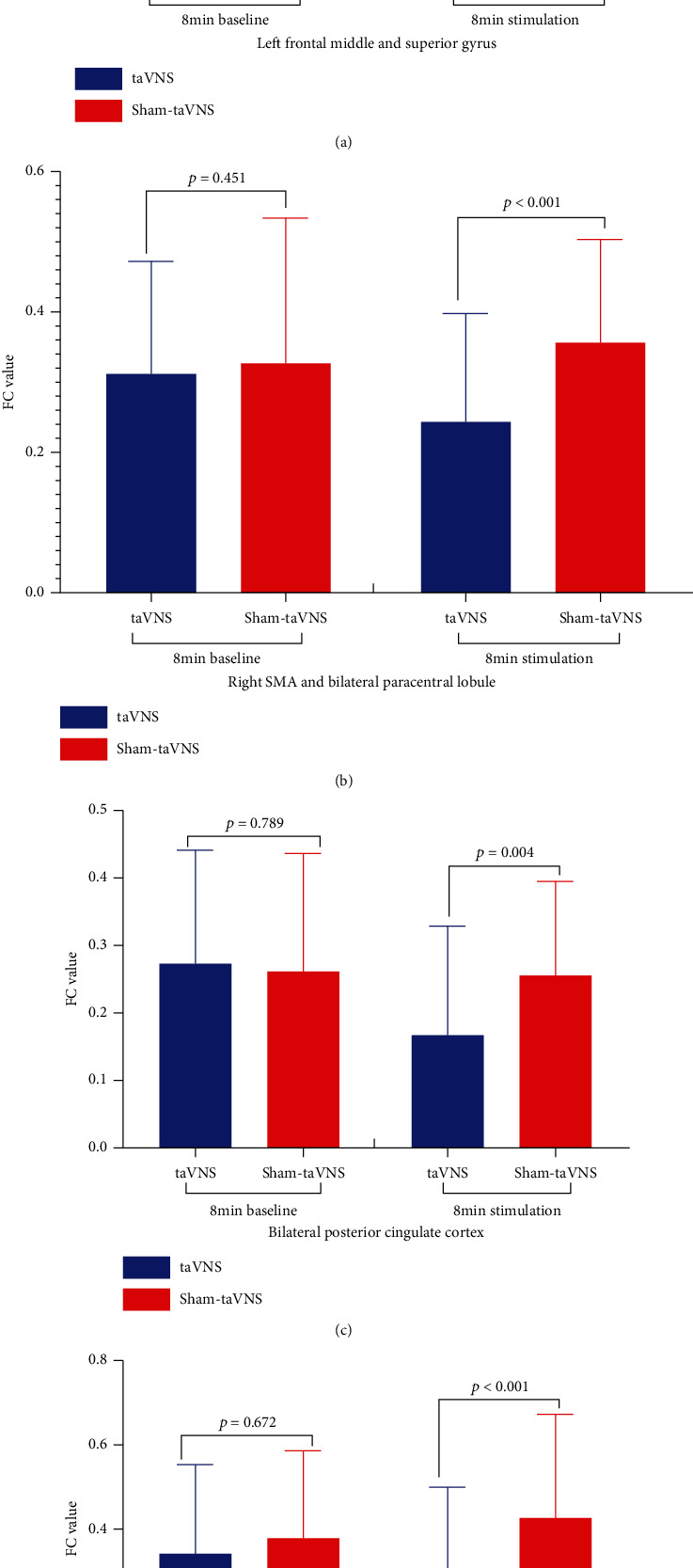
The effects on the functional connectivity (FC) of the left amygdala between stimulations and time factors. SMA: supplementary motor area. A paired *t*-test was used. If *p* < 0.05, the difference was statistically significant. Before stimulation, there was no statistical significance for the FC of (a) the left amygdala and left MFG and SFG, (b) right SMA and paracentral lobule, (c) posterior cingulate cortex, (d) and frontal superior medial gyrus (taVNS versus staVNS). During stimulation, their differences were significant. The intensity of the FC decreased in taVNS and increased in staVNS (prestimulation vs. during stimulation). Therefore, the trend of the changed FC was opposite between taVNS and staVNS.

During stimulation, there were statistical significances for the FC in left MFG and SFG, right SMA and bilateral paracentral lobule, PCC, and right medial superior frontal gyrus (taVNS vs. staVNS) (*t* [1, 26] = −6.149, *p* < 0.001, Figure 4(a); *t* [1, 26] = −4.143, *p* < 0.001, Figure 4(b); *t* [1, 26] = −3.157, *p* < 0.001, Figure 4(c); *t* [1, 26] = −4.734, *p* < 0.001, Figure 4(d)), while there was no significant difference before stimulation (*t* [1, 26] = −1.134, *p* = 0.267, Figure 4(a); *t* [1, 26] = −0.765, *p* = 0.451, Figure 4(b); *t* [1, 26] = 0.271, *p* = 0.789, Figure 4(c); *t* [1, 26] = −0.428, *p* = 0.672, Figure 4(d)) (Supplementary table 1). All the changing trend of these FC were opposite between taVNS and staVNS.

**Figure 5:**
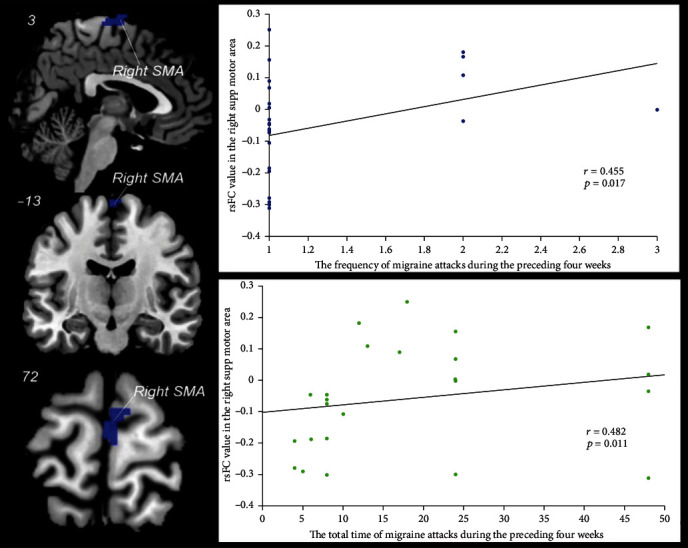
Correlation analysis between migraine attacks and the functional connectivity (FC) of the left amygdala during taVNS. SMA: supplementary motor area; rsFC: resting-state FC; supp: superior. The FC between the left amygdala and the right SMA was correlated with the frequency (*p* = 0.017 and *r* = 0.455) and the total time (*p* = 0.011 and *r* = 0.482) of migraine attacks during the preceding four weeks.

The FC between the left amygdala and right SMA was correlated with the frequency (*p* = 0.017 and *r* = 0.455) and the total time (*p* = 0.011 and *r* = 0.482) of migraine attacks during the preceding four weeks before treatment (Supplementary table 2 and Figure 5), and there was no significant association for other FCs.

#### 3.2.2. Right Amygdala as Seed

**Figure 6:**
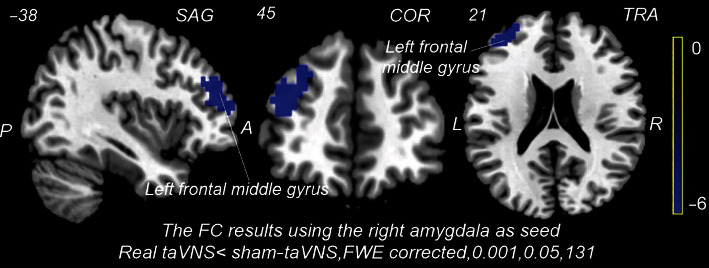
The functional connectivity (FC) of the right amygdala in MwoA during continuous stimulation (taVNS vs. staVNS). SAG: sagittal; COR: coronal; TRA: transverse; FWE: familywise error correction. Compared with staVNS, the FC of the right amygdala and left frontal middle gyrus significantly decreased during taVNS (voxel-level *p* < 0.001 and cluster-level *p*_FWE_ < 0.05).

Compared with the staVNS, the FC significantly decreased between the right amygdala and left MFG in continuous taVNS (Table 3 and Figure 6). The value of the FC between the right amygdala and left MFG does not correlate with the migraine attacks.

**Figure 7:**
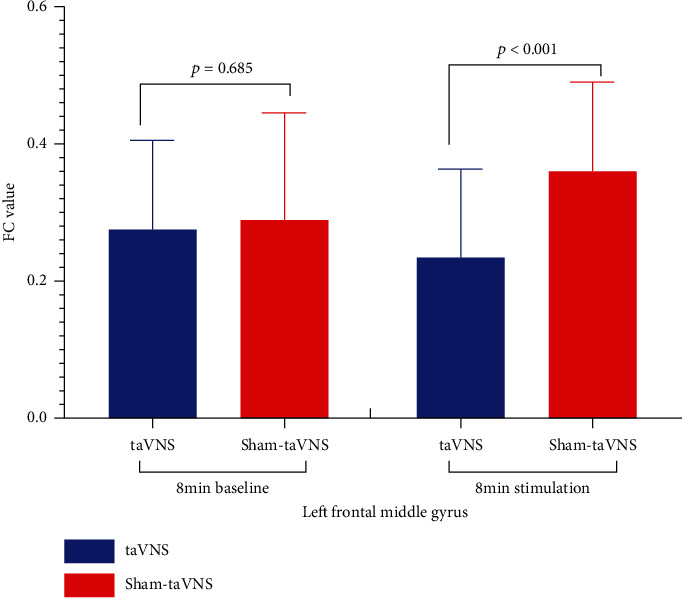
The functional connectivity (FC) comparisons of the right amygdala in different states and stimulations. A paired *t*-test was used. If *p* < 0.05, the difference was statistically significant. Before stimulation, there was no statistical significance for the FC of the right amygdala and left middle frontal gyrus (taVNS vs. staVNS), but the difference was significant during stimulation. The FC decreased in taVNS and increased in staVNS (prestimulation vs. during stimulation). Therefore, the changing trend of the FC was opposite between taVNS and staVNS.

The FC of the right amygdala and the left middle frontal gyrus had no significant difference before stimulation (*t* [1, 26] = −0.410 and *p* = 0.685), with significant difference during stimulation (*t* [1, 26] = −5.789 and *p* < 0.001) (taVNS vs. staVNS) (Supplementary table 3 and Figure 7). The changing trend of the FC was opposite between taVNS and staVNS.

## 4. Discussion

In this trial, we explored the altered FC of the bilateral amygdalae in MwoA during continuous taVNS and staVNS. To summarize, we found that the FCs significantly decreased during taVNS compared with staVNS between the amygdala and DMN (bilateral PCC), prefrontal cortex (PFC: left dorsolateral SFG, left MFG, and right superior medial frontal gyrus), pain matrix (right SMA), and sensorimotor network (bilateral paracentral lobule), with different changes in the FC between the left amygdala and the right amygdala. Moreover, the value of the FC between the left amygdala and right SMA was positively correlated with the frequency and total time of migraine attacks during the preceding four weeks before treatment. Our research suggests that taVNS at auricular points may modulate the function of the limbic system and pain-related networks via adjusting abnormal FC of the amygdalae to treat migraines. We were the first to report the FC of the bilateral amygdalae during continuous taVNS at acupoints and staVNS in MwoA.

The amygdala played a crucial role in the pathogenesis, chronicity, and recurrence of migraines [24, 34], not only in emotion modulation but also in pain processing. Firstly, animal experiments have proved that the amygdala played an essential role in the regulation of synaptic transmission of neurons related to cortical spreading depression (CSD) and the neuropathic pain in migraine [35]. Secondly, migraine patients are often accompanied by negative emotions such as aversion, anxiety, fear, and avoiding pain behavior, which are closely related to the function of the amygdala [26, 28]. Finally, there are a large number of fibrous connections and the FC between the amygdala and pain-related brain regions [24] and networks [21]. Therefore, modulating the dysfunctional FC of the amygdala might be a potential treatment mechanism in MwoA. Meanwhile, our recent study demonstrated that taVNS can modulate the resting-state FC between the bilateral LC and left amygdala and certain pain-related brain regions consistent with the vagus nerve central projections [25]. So, exploring the changed FC of the amygdala in the MwoA during continuous stimulation helps us to understand the mechanism and intervention effects of taVNS.

Antinociceptive effects of the taVNS were demonstrated in numerous animal experiments [36, 37] and clinical studies [38–40]. The taVNS may reduce nociception and pain through multiple mechanisms [41, 42], such as adjusting the autonomic nervous system, suppressing pain neurons, affecting the behavioral pain response, and modulating the pain networks. Reducing the sympathetic hyperactivity and increasing the parasympathetic activity may help manage pain via taVNS [43], with its anti-inflammatory effect together [44]. In animal models, VNS could inhibite nociceptive activation of trigeminal cervical neurons [45] and Fos protein expression [46]. In addition, Pena found that the VNS facilitated the extinction of conditioned fear responses by promoting plasticity of the amygdala and infralimbic area [47]. Furthermore, its effects on the pain networks will be discussed in detail below.

The most interesting finding was that taVNS is capable of regulating the endogenous analgesia loop and descending pain inhibitory system. The FCs decreased between the amygdala and SFG, MFG, and superior medial frontal gyrus during continuous taVNS. This is consistent with the observation from our previous block-designed study that taVNS produced widespread fMRI signal decreased in the bilateral SFG, MFG, and medial prefrontal gyrus on MwoA [29]. As we all know, the medial frontal gyrus belongs to the medial prefrontal cortex, a key node of the endogenous analgesia loop, and descending pain inhibitory system [19]. Meanwhile, the SFG and MFG are parts of the prefrontal cortex which is of great importance for pain perception and response [28]. Both the amygdala and prefrontal cortex were essential for the limbic system widely connected with other nervous systems and participated in pain and emotional regulation. Neuroimaging studies [24] have reported that the FCs were damaged in migraine between the limbic system and pain-related brain areas. Some researchers [19] even put forward the neurological dysfunction model of the limbic pain network in migraine, highlighting the importance of the limbic system in the pathological mechanism of migraine. Notably, migraine patients have abnormal increases in FC within and around the limbic system. For example, Wei and colleagues [48] found that the FC of the limbic system (bilateral amygdala and right hippocampus) and left middle occipital gyrus (MOG) significantly increased in MwoA patients compared with healthy controls, and the FC between the left amygdala and MOG was positively correlated with the duration of migraine. Therefore, limbic system dysfunction plays an important role in the occurrence, development, and regulation of migraines. Further study of acupuncture analgesia [49] found that the amplitude of low-frequency fluctuation (ALFF) in left insula decreased in patients with chronic low back pain after acupuncture, and the decrease of average ALFF was positively related to the decrease of VAS value, which confirms that regulating the function of limbic system may be one of the mechanisms for endogenous analgesia. The taVNS could regulate amygdala activity from a recent study [50] and caused extensive negative activation in the limbic system [30]. Therefore, we speculated that taVNS may modulate the function of the limbic system through the amygdalae, regulating the pain networks, and exerting an analgesic effect.

Another important finding was that DMN is crucial to migraine and can be affected by taVNS. The FC decreased between the left amygdala and bilateral PCC after taVNS compared with staVNS in our current trial. Similarly, we found that taVNS decreased the fMRI signal in the bilateral PCC on MwoA in our previous block-designed study [29]. It is noteworthy that PCC is one core area of DMN closely related to pain perception response [31] and inhibition [51]. Neuroimaging studies [52] have found that headache was associated with a reduced volume of the mPFC. Husoy et al. [24] also found the FC of the left amygdala and DMN increased in MwoA, and the increased FC was associated with the development of headaches. More and more studies confirmed that abnormal FC between the limbic system and DMN was related to pain, so regulating their FC could alleviate pain, which is supported by the results of analgesic treatment research. Zou [53] suggested acupuncture could reduce the FC within DMN to a healthy control level in chronic migraine patients, and the FC between DMN and limbic system also decreased in chronic pain patients after cognitive behavioral therapy [54]. Furthermore, Fang [55] found that compared with staVNS, the FC decreased between the DMN and limbic system after one-month taVNS, suggesting that taVNS can regulate the FC between the limbic system and DMN. Hence, taVNS is a valuable choice for pain treatment.

Last but not least, taVNS can modulate the pain matrix. Our result indicated that the FC between the left amygdala and right SMA decreased after taVNS. It is generally accepted that the SMA contributes to the pain matrix. Imaging studies [56] found that there were functional connections between the amygdala and SMA, indicating that the function of the pain matrix was related to the amygdala. The pain matrix and limbic system had high regional homogeneity (ReHo) [57] in pain. Solstrand [58] indicated that the resting-state FC (rsFC) of SMA and the amygdala increased in migraine patients, and the increased rsFC was negatively correlated with migraine frequency, which revealed that the pain matrix and limbic system worked together in pain perception and regulation, and their increased functional activities were closely related to pain. Fortunately, the abnormally increased functional activity of the amygdala and the pain matrix can be reversed by interventions. For example, Shi [57] found extensive negative activation of brain regions of the limbic system and the pain matrix after acupuncture. It is worth noting that the FC between the amygdala and pain matrix decreased after taVNS in our study. Therefore, taVNS can adjust the abnormal FC between the limbic system and the pain matrix in MwoA patients.

However, there are some limitations in our research. Firstly, we only studied the instant effects of taVNS; thus, more studies are needed to evaluate the long-term impact of taVNS. Secondly, although crossover-control design can save the sample size and eliminate the influence of the differences between individuals, it increased the false-positive rate of the experiment, and randomized controlled studies will be considered. Thirdly, we just explored the altered FC using the seed-to-voxel analysis (same to the published article [24]) during taVNS compared with sham-taVNS. It is a very good idea and very important to explore the effects of taVNS on the pain matrix using the ROI-to-ROI analysis in the follow-up study. Finally, the outcome of this small sample study maybe not enough to be extended to the public, so larger samples are needed in the future.

## 5. Conclusions

From the above discussion, we can conclude that continuous taVNS at auricular points can modulate the FC between the bilateral amygdalae and pain-related regions in MwoA, involving the limbic system, DMN, and pain matrix, with obvious differences between the left amygdala and the right amygdala. The taVNS may produce treatment effects by modulating the abnormal FC of the amygdala and pain networks, possibly having the same central mechanism as auricular acupuncture.

## Figures and Tables

**Table 1:** The demographic and clinical data during the past four weeks (*n* = 27).

Characteristics	(Frequency/mean ± SD)
Gender (male/female)	2/25
Age	29.85 ± 8.09
Disease duration in years	9.22 ± 7.26
Frequency per month	1.22 ± 0.51
Total time per month	18.93 ± 15.61
VAS	42.99 ± 17.01
MSQ	72.52 ± 9.41
SDS score	45.96 ± 9.69
SAS score	42.63 ± 9.87

VAS: Visual Analog Scale; MSQ: Migraine Specific Quality-of-Life Questionnaire; SDS: Self-rating Depression Scale; SAS: Self-rating Anxiety Scale. Frequency/total time per month: The frequency/the total time of migraine attacks during the past four weeks.

**Table 2:** The functional connectivity results using the left amygdala as a seed in 27 MwoA patients.

Contrast	Cluster	Brain region	Peak *T* value	Peak *Z* value	MNI coordinates		
					*X*	*Y*	*Z*
Real>sham	No brain region above the threshold						
Real<sham	178	Left middle frontal gyrus	6.10	4.73	-27	48	30
	68	Left superior frontal gyrus	5.22	4.25	-21	60	6
	18	Right supplementary motor area	5.37	4.34	3	-9	72
	18	Bilateral paracentral lobule	5.00	4.12	-3	-21	75
	17	Bilateral post cingulum gyrus^∗^	4.47	3.80	3	-51	27
	15	Right frontal superior medial gyrus^∗^	4.59	3.87	3	63	0

^∗^Small-volume correction at *p*_FWE_ < 0.001.

**Table 3:** The functional connectivity results using the right amygdala as a seed in 27 MwoA patients.

Contrast	Cluster	Brain region	Peak *T* value	Peak *Z* value	MNI coordinates		
					*X*	*Y*	*Z*
Real>sham	No brain region above the threshold						
Real<sham	131	Left middle frontal gyrus	5.54	4.43	-38	45	21

## Data Availability

The data (fMRI) used to support the findings of this study are included within the article and the supplementary information files.

## Supplementary Materials

Supplementary Materials Supplementary Table 1: the functional connectivity comparisons of the left amygdala in different states and stimulations. Supplementary Table 2: correlations of the functional connectivity and clinical data. Supplementary Table 3: the functional connectivity comparisons of the right amygdala in different states and stimulations.
