# Supplementary material for: The Instant Effects of Continuous Transcutaneous Auricular Vagus Nerve Stimulation at Acupoints on the Functional Connectivity of Amygdala in Migraine without Aura: A Preliminary Study
Source: Neural Plast. 2020 Dec 10;2020:8870589. doi: 10.1155/2020/8870589 (PMC7759401; doi:10.1155/2020/8870589)
Supplement: Supplementary Materials — Supplementary Table 1: the functional connectivity comparisons of the left amygdala in different states and stimulations. Supplementary Table 2: correlations of the functional connectivity and clinical data. Supplementary Table 3: the functional connectivity comparisons of the right amygdala in different states and stimulations. [file 8870589.f1.docx]

Supplementary table 1. The functional connectivity comparisons of the left amygdala in different states and stimulations

| **Brain Regions** | **state** | **taVNS** | **staVNS** | **Effect of stimulation** |
| --- | --- | --- | --- | --- |
| L_MFG/SFG | Pre-stim | 0.258 ± 0.113  0.214 ± 0.119 | 0.286 ± 0.108  0.336 ± 0.103 | t (1,26) = -1.134, *p*=0.267 |
|  | Dur-stim |  |  | t (1,26) = -6.149, ***p*<0.001** |
|  |  |  |  |  |
| R_ frontal superior  medial gyrus | Pre-stim | 0.312 ± 0.162 | 0.329 ± 0.205 | t (1,26) = -0.428, *p*=0.672 |
|  | Dur-stim | 0.244 ± 0.153 | 0.357 ± 0.147 | t (1,26) = -4.734, ***p*<0.001** |
|  |  |  |  |  |
| bilateral PCC | Pre-stim | 0.272 ± 0.169 | 0.261± 0.173 | t (1,26) = 0.271, *p*=0.789 |
|  | Dur-stim | 0.166 ± 0.161 | 0.257± 0.138 | t (1,26) = -3.157, ***p*=0.004** |
| R_SMA/  bilateral paracentral lobule | Pre-stim | 0.342 ± 0.211 | 0.379 ± 0.206 | t (1,26) = -0.765, *p*=0.451 |
|  | Dur-stim | 0.255 ± 0.244 | 0.429 ± 0.243 | t (1,26) = -4.143, ***p*<0.001** |

TaVNS: transcutaneous auricular vagus nerve stimulation; staVNS: sham-taVNS; L: left; R: right; MFG: frontal middle gyrus; SFG: dorsolateral superior frontal gyrus; PCC: post cingulum cortex; SMA: supplementary motor area. Pre-stim: pre-stimulation; Dur-stim: during stimulation. A paired t-test was used. If *p*<0.05, the difference was statistically significant. Before stimulation, there was no statistical significance for the functional connectivity (FC) of the left amygdala and left MFG, left SFG, right frontal superior medial gyrus, bilateral PCC, right SMA, and bilateral paracentral lobule between taVNS and staVNS, while the differences were significant during stimulation. The intensity of the FC decreased (pre-stimulation vs during stimulation) with taVNS, while the FC increased with staVNS. The change trend of the FC was opposite between taVNS and staVNS.

Supplementary table 2. Correlations of the functional connectivity and clinical data

| **Brain Regions** | **frequency** | **Total time** | **VAS** |
| --- | --- | --- | --- |
| L_Amy_L_ MFG/SFG | r = -0.014  *p* = 0.946 | r = -0.044  *p* = 0.829 | r = 0.049  *p* = 0.808 |
|  |  |  |  |
| L_Amy_R_frontal superior medial gyrus | r = -0.174  *p* = 0.387 | r = 0.038  *p* = 0.853 | r = -0.106  *p* = 0.598 |
|  |  |  |  |
| L_Amy_bilateral PCC | r = -0.005  *p* = 0.980 | r = 0.091  *p* = 0.652 | r = 0.101  *p* = 0.615 |
|  |  |  |  |
| L_Amy_R_SMA  L_Amy_bilateral paracentral lobule | **r = 0.455**  ***p* = 0.017**  r = 0.385  *p* = 0.047 | **r = 0.482**  ***p* = 0.011**  r = 0.409  *p* = 0.034 | r = 0.329  *p* = 0.093  r = 0.123  *p* = 0.541 |
|  |  |  |  |
| R_Amy_L_MFG | r = -0.269  *p* = 0.174 | r = -0.262  *p* = 0.188 | r = 0.040  *p* = 0.843 |

L: left; R: right; Amy: amygdala; MFG: middle frontal gyrus; SFG: superior frontal gyrus; PCC: post cingulum cortex; SMA: supplementary motor area. Spearman’s correlation was used. If *p*<0.05, the difference was statistically significant. The FC between the left amygdala and right SMA was correlated with the frequency (*p* = 0.017, r = 0.455) and the total time (*p* = 0.011, r = 0.482) of migraine attacks during the preceding four weeks before treatment.

Supplementary table 3. The functional connectivity comparisons of the right amygdala in different states and stimulations

| **Brain Regions** | **state** | **taVNS** | **staVNS** | **Effect of stimulation** |
| --- | --- | --- | --- | --- |
| Left MFG | Pre-stim | 0.276 ± 0.129  0.235 ± 0.127 | 0.289 ± 0.155  0.360 ± 0.130 | t (1,26) = -0.410, *p*=0.685 |
|  | Dur-stim |  |  | t (1,26) = -5.789, ***p*<0.001** |

TaVNS: transcutaneous auricular vagus nerve stimulation; staVNS: sham-taVNS; MFG: middle frontal gyrus; Pre-stim: pre-stimulation; Dur-stim: during stimulation. A paired t-test was used. If *p*<0.05, the difference was statistically significant. Before stimulation, there was no statistical significance for the functional connectivity (FC) of the right amygdala and left MFG between taVNS and staVNS, but the difference was significant during stimulation. The intensity of the FC decreased (pre-stimulation vs during stimulation) with taVNS, while the FC increased with staVNS. The change trend of the FC was opposite between taVNS and staVNS.
